# Addition of αGal HyperAcute™ technology to recombinant avian influenza vaccines induces strong low-dose antibody responses

**DOI:** 10.1371/journal.pone.0182683

**Published:** 2017-08-07

**Authors:** Wenlan Alex Chen, Jinjin Zhang, Katie M. Hall, Carol B. Martin, Serguei Kisselev, Emily J. Dasen, Nicholas N. Vahanian, Charles J. Link, Brian K. Martin

**Affiliations:** NewLink Genetics Corp., Ames, Iowa, United States of America; Sun Yat-Sen University, CHINA

## Abstract

Highly pathogenic avian influenza represents a severe public health threat. Over the last decade, the demand for highly efficacious vaccines against avian influenza viruses has grown, especially after the 2013 H7N9 outbreak in China that resulted in over 600 human cases with over 200 deaths. Currently, there are several H5N1 and H7N9 influenza vaccines in clinical trials, all of which employ traditional oil-in-water adjuvants due to the poor immunogenicity of avian influenza virus antigens. In this study, we developed potent recombinant avian influenza vaccine candidates using HyperAcute^™^ Technology, which takes advantage of naturally-acquired anti-αGal immunity in humans. We successfully generated αGal-positive recombinant protein and virus-like particle vaccine candidates of H5N1 and H7N9 influenza strains using either biological or our novel CarboLink chemical αGal modification techniques. Strikingly, two doses of 100 ng αGal-modified vaccine, with no traditional adjuvant, was able to induce a much stronger humoral response in αGT BALB/c knockout mice (the only experimental system readily available for testing αGal *in vivo*) than unmodified vaccines even at 10-fold higher dose (1000 ng/dose). Our data strongly suggest that αGal modification significantly enhances the humoral immunogenicity of the recombinant influenza vaccine candidates. Use of αGal HyperAcute^™^ technology allows significant dose-sparing while retaining desired immunogenicity. Our success in the development of highly potent H5N1 and H7N9 vaccine candidates demonstrated the potential of αGal HyperAcute^™^ technology for the development of vaccines against other infectious diseases.

## Introduction

Vaccination has been a common strategy for preventing seasonal influenza worldwide over the last several decades [1]. Current seasonal influenza vaccines include live-attenuated influenza vaccines (LAIV) such as FluMist^®^, trivalent inactivated influenza vaccines (TIV) e.g. Fluzone^®^, and the newly approved recombinant vaccine Flublok^®^. However, these vaccines do not elicit strong protection for pandemic influenza viruses, especially in the case of H5N1 and H7N9 avian influenza. For reasons that are not clear, avian influenza hemagglutinin (HA) proteins are less immunogenic compared with HAs of seasonal influenza strains. Most seasonal vaccinations occur in the context of pre-existing virus immunity, due to prior exposure to seasonal influenza strains through infection and/or previous vaccinations. In contrast, most humans have never been exposed to pandemic zoonotic influenza strains. This difference suggests that zoonotic influenza antigens are less immunogenic compared with HAs of seasonal influenza strains in the context of pre-existing immunity. In order to improve the vaccine efficacy, adjuvants have become necessary in the formulation of these vaccines. To date, the adjuvant system 03 (AS03) developed by GlaxoSmithKline (GSK) has been conditionally approved for monovalent H5N1 vaccine for the U.S. stockpile in case of pandemic outbreak [2]. In addition, oil-in-water emulsion MF59^®^ (Novartis) adjuvanted seasonal influenza vaccine, FLUAD^®^ has been licensed for older adults in Europe since 1997. Meanwhile, H7N9 vaccines formulated with either AS03, or the MF59 adjuvants, are under Phase I and II clinical evaluations [3] (Clinical Trials NCT01938742, NCT01942265, and NCT02177734).

**Fig 1:**
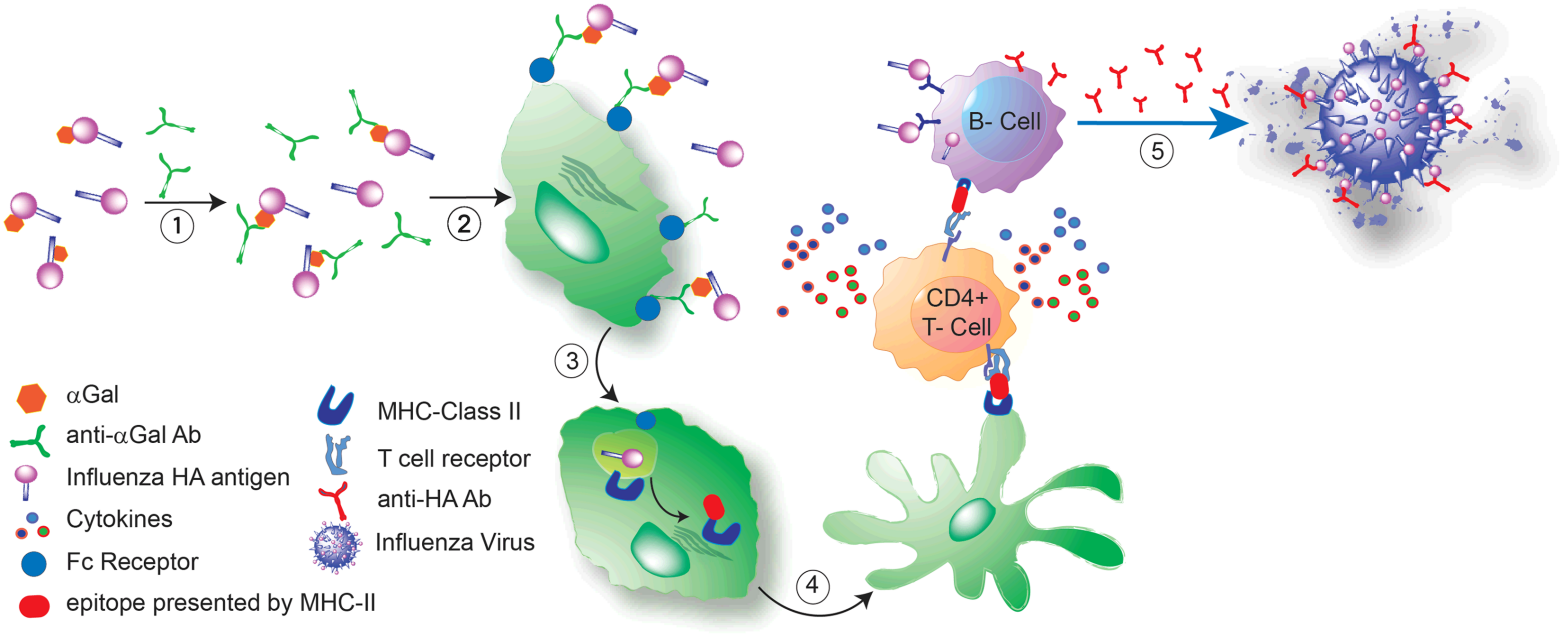
Illustration of HyperAcute^™^ vaccine design leading to enhanced immunogenicity. (1) Immune complex formation between pre-existing anti-αGal antibody and αGal modified influenza HA antigen. (2) Interaction between Fcγ on APC and the Fc from the immune complex. (3) Enhancement of HA uptake by APC via the Fcγ-Fc interaction. (4) Presentation of HA epitope by MHC-II molecule to CD4+ T cells, and consequent stimulation of HA specific B cells. (5) Significant anti-HA antibody production leads to protection from influenza virus infection *via* humoral immunity.

In addition to the traditional oil-in-water type adjuvants mentioned above, there are several other types of adjuvants that have been approved [4–6] or are in preclinical/clinical evaluation [7–10] for use in infectious diseases and cancer treatment. Recently, natural adjuvants based on immune complexes between antibodies and specific antigens have been intensively examined[11]. αGal HyperAcute^™^ Technology is an example of this new class of natural adjuvants for clinical applications[12–15]. αGal HyperAcute^™^ technology exploits a robust zoonotic blockade against viruses from lower animals to enhance potency of antiviral vaccines (Fig 1). Naturally-acquired immunity against the common αGal epitope (Gal-α1,3-Gal-β1,4-GlcNAc-R or Gal-α1,3-Gal-β1,4-Glc-R) in humans is facilitated by the lack of a functional gene for the enzyme α1,3-galactosyltransferase (αGT), in the N-glycosylation biosynthetic pathway [16]. Since human cells do not have this epitope, chronic stimulation from gut flora leads to high levels of circulating anti-αGal antibodies and the development of a robust immune response [17, 18]. Since αGal epitopes can be immediately recognized by circulating antibodies, the natural αGal immune pathway could serve as a barrier against zoonotic infection in humans, including viruses that come from lower mammals which have αGal epitopes on their lipids (in the case of enveloped viruses) and glycoproteins. Because all mammals except Old-World monkeys have a functional αGT enzyme, knockout models (especially the knockout mouse) are used to test the effect of αGal on immunogenicity. The αGal HyperAcute^™^ technology takes advantage of this natural process to stimulate the rapid and efficient presentation of modified antigens to antigen-presenting cells (APCs) [19], leading to a strong immune response against target antigens.

Unlike traditional adjuvants, the αGal epitope not only has adjuvant-like activity but also simultaneously acts as an antigen itself. This technology can be utilized to exploit pre-existing immunity to αGal in human in order to mount a strong immune response against antigens, such as influenza virus antigens. Furthermore, because αGal technology directly conjugates αGal to antigens of interest, it obviates formulation and QA/QC issues encountered with other adjuvants that must be added to vaccines subsequent to manufacturing.

In this study, two different αGal modification strategies have been successfully developed and applied to H5N1 and H7N9 influenza strains to produce monovalent recombinant HA protein and virus like particle (VLP) vaccine candidates. The immunogenicity of these αGal-modified vaccine candidates was evaluated in an αGT knockout mouse model. The results indicate that αGal-modification of vaccines can enhance efficacy and protective immunity against influenza virus. Addition of αGal to vaccine antigens, such as influenza virus antigens, has the potential to allow dose-sparing of current vaccines or improve immunogenicity of vaccines under development.

## Materials and methods

### Cloning

To make recombinant influenza hemagglutinin (H5N1 HA GenBank: AET80428.1, or H7N9 HA GenBank: AGI60301.1), a construct was produced to include a DNA sequence encoding a 5’ IgG-kappa signal peptide (METDTLLLWVLLLWVPGSTG) [20], the HA gene with the transmembrane domain deleted, a 3’ fusion domain containing a thrombin cleavage sequence (LVPRG) [21], a GCN4 trimerization motif (RMKQIEDKIEEILSKIYHIENEIARIKKLVGER) [22–24], and a 6XHis tag. This construct was synthesized by Genscript and cloned into the pCAGGS vector using EcoRI and XhoI restriction sites. To make recombinant influenza VLPs, the full length sequence of HA gene (H5N1 HA GenBank: AET80428.1, or H7N9 HA GenBank: AGI60301.1), NA (H5N1 NA GenBank: AET80428.1, or H7N9 NA GenBank: AGI60300.1), and M1 (H7N9 M1 GenBank YP_009118478.1) were cloned into the pCAGGS vector. Marmoset αGT (GenBank: NP_001254661.1) was cloned into the pcDNA3.1 vector.

### Recombinant protein expression and purification

The details of the methods used to express and purify both recombinant protein and VLP vaccines can be found in the S1 File.

### Chemical modification of vaccine candidates

The details of the methods used to chemically modify the vaccine candidates can be found in the S2 File.

### Process of pig kidney membrane for αGal priming

Adult pig kidneys were purchased from Pel-Freez Biologicals (AR, USA) and irradiated at a dose of 25 kGy at Sadex Radiation Processing Center (IA, USA). Irradiation was necessary as standard kidneys from meat processing plants have intracellular bacteria that can be transferred to mice (data not shown). Irradiated kidneys were minced, homogenized with PBS and aliquots of the homogenate at 100 mg/ml were stored at -20°C. All work related to pig kidney membrane (PKM) processing was performed in a biosafety cabinet and all materials that were in direct contact with PKM were autoclaved. The processed PKM was 10-fold serially diluted and inoculated on tryptic soy agar plates. None of the plates had any colonies and the material passed the sterility test. Prior to each priming, an aliquot of PKM was thawed and formulated with CpG (IDT) and IFA (Sigma, F5506).

### Mouse vaccination

All animal experiments were conducted at Laboratory Animal Resources (LAR) at Iowa State University (ISU), and the protocols were approved by Institutional Animal Care and Use Committee (IACUC) at ISU before study initiation. The mice were housed using the Innovive cage system with ventilated racks on a 12 hour light-dark cycle. Mice were housed at four animals per cage with weekly cage changes with unlimited access to sterilized food and water. Enrichment consisted of the use of nesting material.

Female αGal knockout BALB/c mice [25, 26], aged 8–12 weeks (18–24 g weight) were maintained as a breeding colony by NewLink Genetics at the ISUA LAR facility. These animals were primed with three intraperitoneal injections of 10 mg PKM, 2.5 μg CpG, and 50 μL IFA at 14-day intervals. Mouse sera were collected to analyze αGal antibody level by ELISA, and mice were grouped (n = 12) such that all vaccination groups had similar anti-αGal titers (including the control group that received injection of diluent only). The group size was determined by examining the standard deviation of previously published research and calculating the number of animals that would give a power of 0.8. Then mice were immunized subcutaneously with the influenza vaccine candidates with or without adjuvant and were boosted four weeks later with the same dose of immunogens ranging from 100 to 1000 ng. The identity of the groups was not indicated on the cage cards such that assessment of the individuals was done blinded. Adjuvanted vaccines were prepared following the instructions provided by the manufacturer of each adjuvant. Blood was drawn from the saphenous vein one day before the first influenza vaccination and two weeks after the second vaccination. Blood draw was the final outcome and mice were sacrificed after the final draw. Each experiments was repeated at least once.

### Antibody detection by ELISA

Plates (96-well) were coated overnight at 4°C with 50 μL antigen (1 ng/μL HA [from Sino Biological] or 5 ng/μL BSA-αGal [from V-Labs Inc.]) in 50 mM carbonate/bicarbonate buffer (Sigma-Aldrich, C3041). The plates were washed with 1X DPBS with 0.05% Tween 20 (wash buffer) and blocked with 150 μL of 1% BSA in 50 mM carbonate/bicarbonate buffer (blocking buffer). The plates were washed as described above, and 50 μL mouse sera that were serially diluted with 1% BSA in wash buffer were added to each well. After 1 hour incubation at room temperature, the plates were washed, and then 50 μL HRP-conjugated goat anti-mouse IgG Fc-γ (Jackson ImmunoResearch, 115-035-071, 1:20,000 diluted in wash buffer) was added to each well. Following a 30-minute incubation at room temperature with the secondary antibody, the plates were washed and then 100 μL peroxidase substrate (KPL, 50-76-00) was added to each well. The reaction continued for 10 minutes and then was stopped by adding 100 μL of 2M sulfuric acid. The absorbance was measured at 450 nm using EPOCH plate reader (BioTek) with Gen5 software, and the optical density (OD) values were graphed using GraphPad Prism V6.05. Statistics analysis results are presented as mean with SEM. *p < 0.05, **p < 0.01, ***p < 0.001, ****p < 0.0001 for αGal positive versus αGal negative vaccines (by unpaired Student *t* test).

### Antibody endpoint titer determination by ELISA

Endpoint ELISA titers were determined by following the same ELISA protocol for antibody detection, with serum dilutions extending to 25,600 fold. The absorbance was measured at 450 nm, and the optical density (OD) values were graphed using GraphPad Prism V6.05. The endpoint OD cutoff was defined as 3 times of the average background OD value. For example, if the background OD was 0.05, then the endpoint OD cutoff is 0.15. For the samples whose first dilutions were below OD 0.15, their endpoint titers were defined as one dilution up. For instance, the endpoint titer of a sample with OD 0.10 at the first dilution (1:100) was defined at 50. Statistics analysis results are presented as mean with SEM. *p < 0.05, **p < 0.01, ***p < 0.001, ****p < 0.0001 for αGal positive versus αGal negative vaccines (by unpaired Student *t* test).

### ImmunoGold staining of H7N9 VLPs and Electron Microscopy

H7N9 VLPs (4 μL; 300 ng/μL) were first incubated on a copper EM grid for 2 minutes. The grid was blocked in a 40 μL drop of 1X DPBS supplemented with 5% BSA (AURION BSA-c, 900.099) and 0.1% cold water fish skin gelatin (AURION, 900.033). Following a 30-minute incubation in a glass petri dish incubation chamber, the grid was stained in a 40 μL drop of rabbit polyclonal anti-HA7 antibody (Sino Biological Inc., 40103-RP02, 1: 500 dilution in blocking buffer) or blocking buffer alone to serve as a negative control. The grid was placed in a 40 μL drop of secondary antibody (Donkey-anti Rabbit IgG, Gold 10nm particle size, Electron Microscopy Sciences, 2705; diluted 1:40 in blocking buffer). The grids were stained with 2% uranyl acetate and observed using a 200kV JEOL 2100 scanning and transmission electron microscope (Japan Electron Optics Laboratories). Images were captured using a high-resolution digital camera (U-1000, www.gatan.com).

### Hemagglutination assay

Hemagglutination assay was performed essentially as previously described [27]. Briefly, 1X DPBS (50 μL) was added to each well of a 96-well U-bottom plate. The purified HA protein (1000 ng in 50 μL) was added to the first well of a row followed by a two-fold dilution across the plate. Turkey red blood cells (Lampire Bio Lab, Cat#7249408), 50 μL of a 0.5% solution prepared in 1X DPBS, were added to each well and mixed gently. The plate was incubated at 4°C for 2 hours before the titer was read and recorded. The end-point titer was defined as the highest dilution of the protein that produced a positive result, indicated by the complete absence of settled turkey red blood cells at the bottom of the well.

### Hemagglutination inhibition (HI) assay

HI assays were performed as previously described [28]. Four (4) HA Unit/25 μL of either influenza H5N1 or H7N9 HA antigens were used as control antigen, and 0.5% turkey red blood cell (Lampire Bio Lab, 7249408) were used. To remove nonspecific inhibitors, all mouse sera were heat inactivated at 56°C for 30 minutes. The hemagglutination activity (4 HA Unit/25 μL) of control antigen was always confirmed on the same day that all samples were analyzed. Titers less than 10 were assigned a value of 0 for calculation purpose. HI titers were graphed using GraphPad Prism V6.05. Statistics analysis results are presented as mean with SEM. *p < 0.05, **p < 0.01, ***p < 0.001, ****p < 0.0001 for αGal positive versus αGal negative vaccines (by unpaired Student *t* test).

### Sialic acid quantification assay

Recombinant HA proteins were treated with neuraminidase from *C*. *perfringens* (Sigma, N2876) for 16 hours at 37°C to release sialic acid as free N-acetylneuraminic acid (NANA). NANA was quantified using Sialic Acid Assay kit (abcam, ab83375) with the fluorometric assay protocol according to the manufactures instructions. In brief, neuraminidase-treated recombinant proteins and provided NANA standards were incubated with sialic acid reaction mixture at 37°C for 30 minutes, and then fluorescence was measured using SpectraMax Gemini microplate reader (Molecular Device) at Ex/Em 535/587. Sialic acid concentrations of recombinant proteins were calculated using NANA standard curve (nmole/well). Numbers of sialic acid per molecule of protein were determined for each recombinant protein taking into account the concentration of protein in each reaction mixture as well as the molecular weight of each protein.

## Results

### Development and optimization of αGal modification strategies

**Fig 2:**
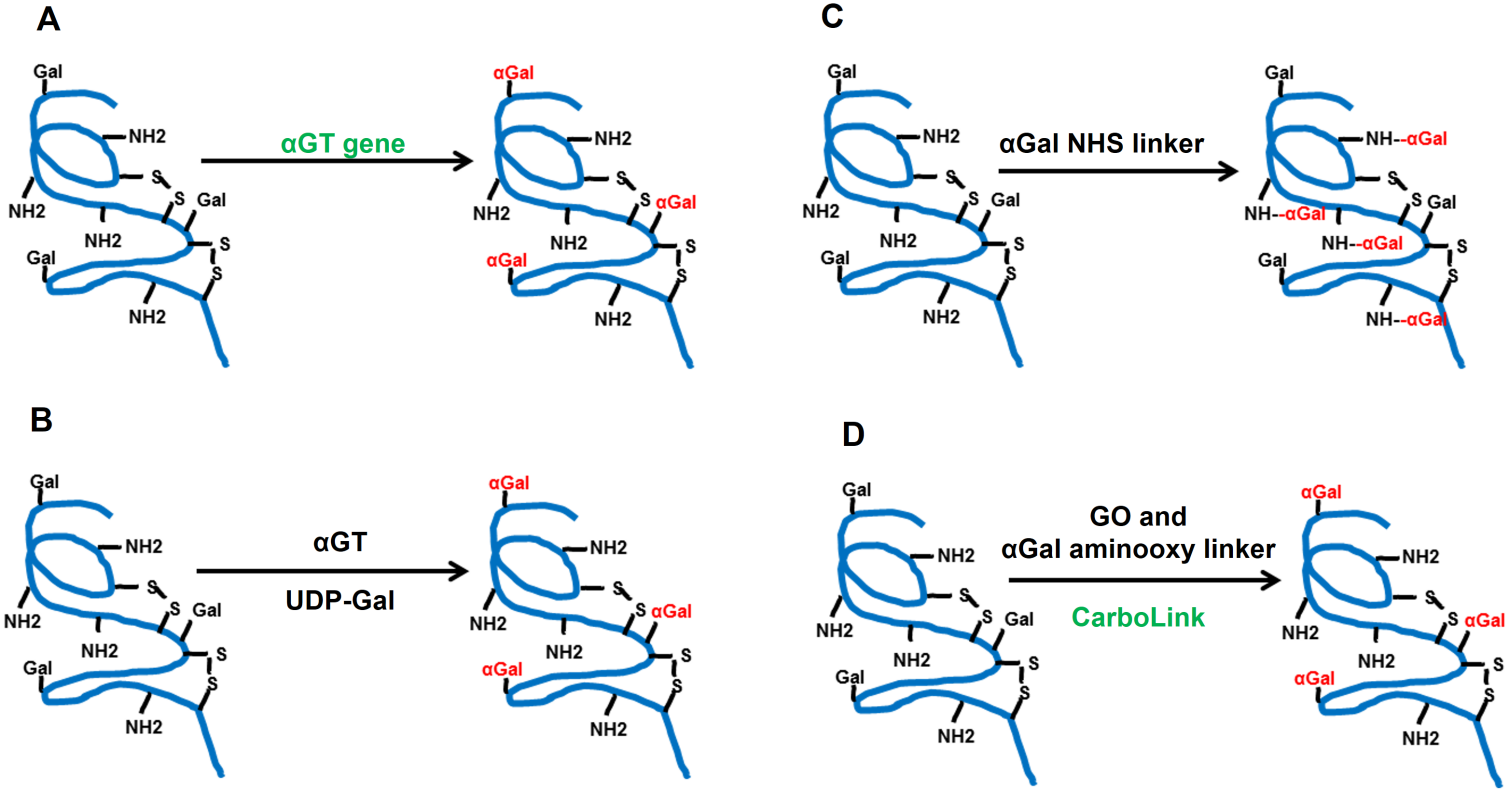
Different αGal modification strategies. (A) αGal biological modification using αGT, (B) Enzymatic modification using αGT and UPD-Gal, (C) Chemical modification using αGal NHS-activated linker, and (D) Chemical (CarboLink) modification using GO and αGal aminooxy linker. (GO: galactose oxidase; Gal: galactose; αGal: Gal-α1,3-Gal-R; NH_2_: lysine residue; S-S: disulfide bond; αGT: α1,3-Galactosyltransferase)

In order to investigate potential of the αGal HyperAcute^™^ technology on influenza vaccines, we developed strategies to conjugate αGal epitope on vaccine candidates, including recombinant proteins, virus-like particles (VLP) and inactivated virus. Our current biological αGal modification strategy adds αGal molecules on recombinant HA proteins by co-transfecting genes of αGT and HA (Fig 2A). The αGT enzyme catalyzes the formation of Gal-α1,3-Gal-β1,4-GlcNAc-R (one type of αGal epitope) [29], by transferring a galactose molecule to an existing terminal galactose predominantly on N-glycosylation sites *via* a specific terminal α1,3-O-glycosidic linkage. The αGT gene co-expression strategy is superior to αGT enzymatic modification (Fig 2B) because it avoids the use of the costly and unstable substrate UDP-Gal and purified αGT enzyme. However, this modification strategy is restricted to mammalian-sourced glycoprotein targets which contain N-glycans. For instance, biological αGal modification is not optimal on either egg-derived influenza vaccines, or insect cell-based products, such as Flublok^®^, since their intrinsic N-glycosylation patterns lack the αGT substrate[30], i.e. terminal galactose residues, or the loss of glycosylation site during adaption [31], when compared to mammalian complex N-glycosylation [30]. In other words, alternative αGal-modification technologies are necessary and crucial for broad applications.

Bioconjugation is a powerful technique to potentially overcome this barrier. We initiated αGal bioconjugation using an N-hydroxylsuccinimide ester (NHS) activated αGal linker (Fig 2C), which cross-links an αGal trisaccharide linker to lysine residues on a protein. This method works on protein targets in amine-free physiological media, and the reaction is highly efficient to introduce a large number of αGal molecules on any protein target due to the relatively high amount of lysine residues available. However, this strategy may cause the blockage of biologically active regions due to the broad distribution of lysine residues. In addition, too many modifications on the protein surface, especially on charged residues, could lead to loss of biological activity due to changes in protein surface charge, hydrophilicity/hydrophobicity and conformation. For instance, recombinant hemagglutinin (rHA) proteins modified by this method lost hemagglutination activity (data not shown), which is an important biological activity marker for a functional influenza HA. To solve this problem, we have developed an advanced carbohydrate-specific αGal chemoenzymatic bioconjugation technology (CarboLink) (Fig 2D) [32]. The key design in this novel strategy is the development of a new type of αGal chemical linker which bears an aminooxy (-ONH_2_) functional group. This type of linker readily reacts with any carbonyl (-C = O) functional group on any target. In this design, mild oxidation of the target protein using either sodium periodate solution or galactose oxidase (GO) is performed as the first step, which generates carbonyl functional groups from pre-existing N-linked or O-linked glycans. During the second step of reaction, αGal aminooxy linker transfers the αGal molecules onto the generated carbonyl groups *via* the formations of oxime bond (O-N = C) under slightly acidic conditions. After two αGal modification strategies (Fig 2A and 2D, Methods) were successfully established, αGal HyperAcute^™^ technology was evaluated *in vitro* and *in vivo* on H5N1 and H7N9 influenza vaccine candidates.

### Expression, purification and characterization of rHA proteins

**Fig 3:**
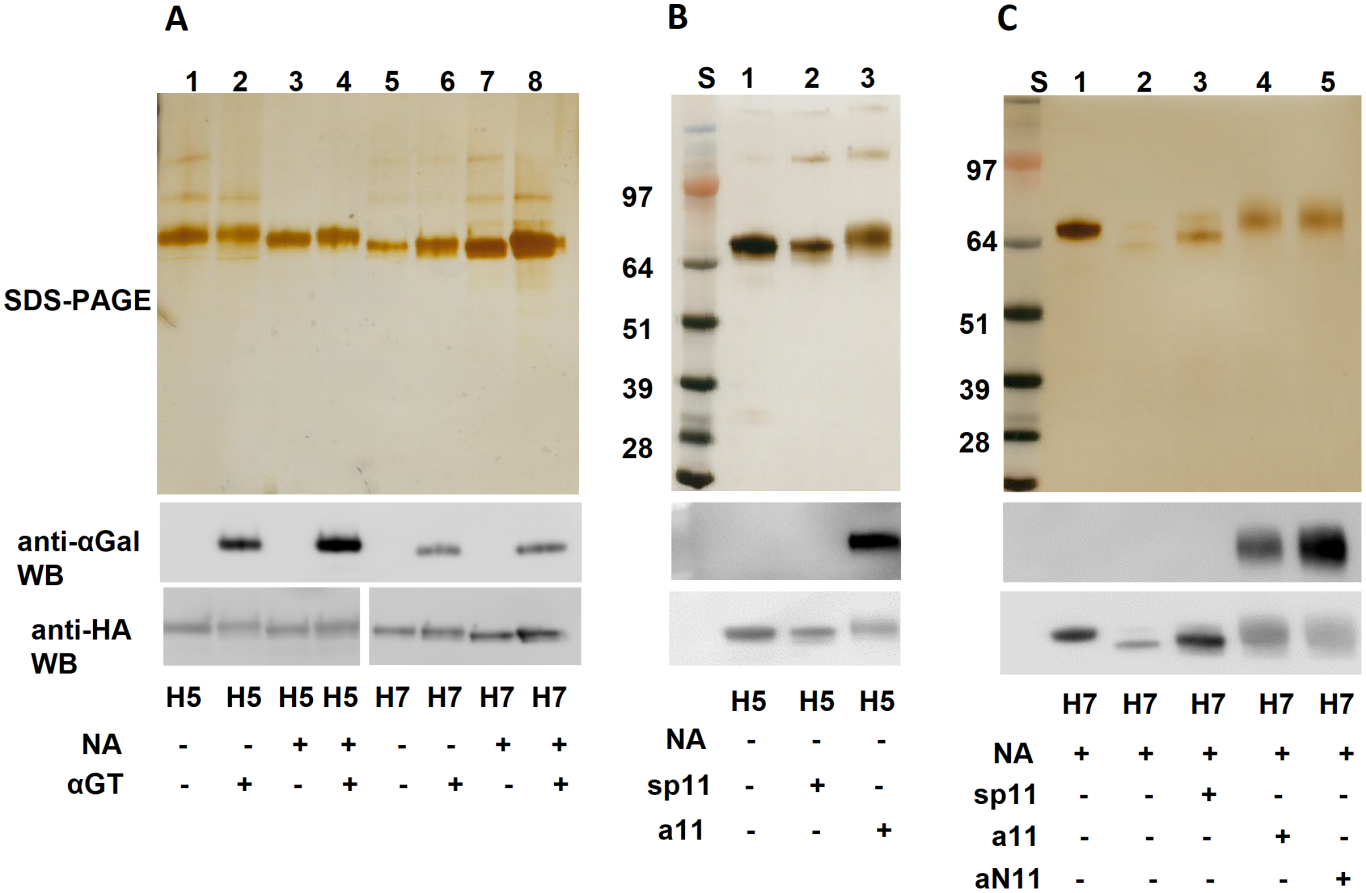
Biological and CarboLink modifications of trimeric rHA vaccine candidates. (A) Biological modification of H5N1 and H7N9 HAs with αGT with or without NA co-expression. (B) Chemical modification of H5N1 HA. (C) Chemical modification of H7N9 HA. Sp11 is the aminooxy spacer control linker without αGal antigen; a11 is one type of αGal (Galα1,3-Galβ1,4-Glc) aminooxy linker; aN11 is the other type of αGal (Galα1,3-Galβ1,4-GlcNAc) aminooxy linker.

The native trimeric conformation of HA proteins plays an important role not only in hemagglutination activity, but also in elicitation of a strong immune response [22, 33]. In order to produce rHA with trimeric structure, we designed HA constructs (including both HA1 and HA2 domains) with an N-terminal IgG-kappa signal peptide and a C-terminal domain consisting of a thrombin cleavage sequence, a GCN4 trimerization motif and a 6xHis tag. For each HA (H5 or H7), four versions of proteins were produced, HA only (HA), HA with neuraminidase (NA^(+)^HA), HA with αGT(αGal^(+)^HA), and HA with both αGT and NA (NA^(+)^αGal^(+)^HA) by transient transfection in mammalian cells (HEK293F). NA is an enzyme that hydrolyzes terminal sialic acids on glycoproteins, which exposes the second last carbohydrate, predominantly galactose on N-glycosylation sites. The αGT enzyme catalyzes the transfer of a galactose to a exposed terminal galactose *via* an α1,3-*O*-glycosidic linkage, generating the αGal epitope on glycosylation sites. Glycosylation composition of a glycoprotein varies in the presence of NA and αGT, which affects the size of the glycoprotein as well as the potential level of αGal modifications that can be made on a glycoprotein *via* their enzymatic activity. As a result, the four different co-transfection schemes produced four proteins of slightly different sizes on a SDS-PAGE (Fig 3A). All HA proteins were confirmed by anti-HA western blot using corresponding antibodies. Compared to HA alone, NA^(+)^HA had slightly reduced molecular weight (slightly faster migrating band compared to HA only) due to the removal of sialic acids. Although there was no distinctive change of molecular weight observed from SDS-PAGE after αGal modification, positive signals in both αGal^(+)^HA and NA^(+)^αGal^(+)^HA sample from anti-αGal western blots indicated that these HA proteins were successfully modified with αGal *via* αGT co-expression.

### Production and characterization of H7N9 VLPs

**Fig 4:**
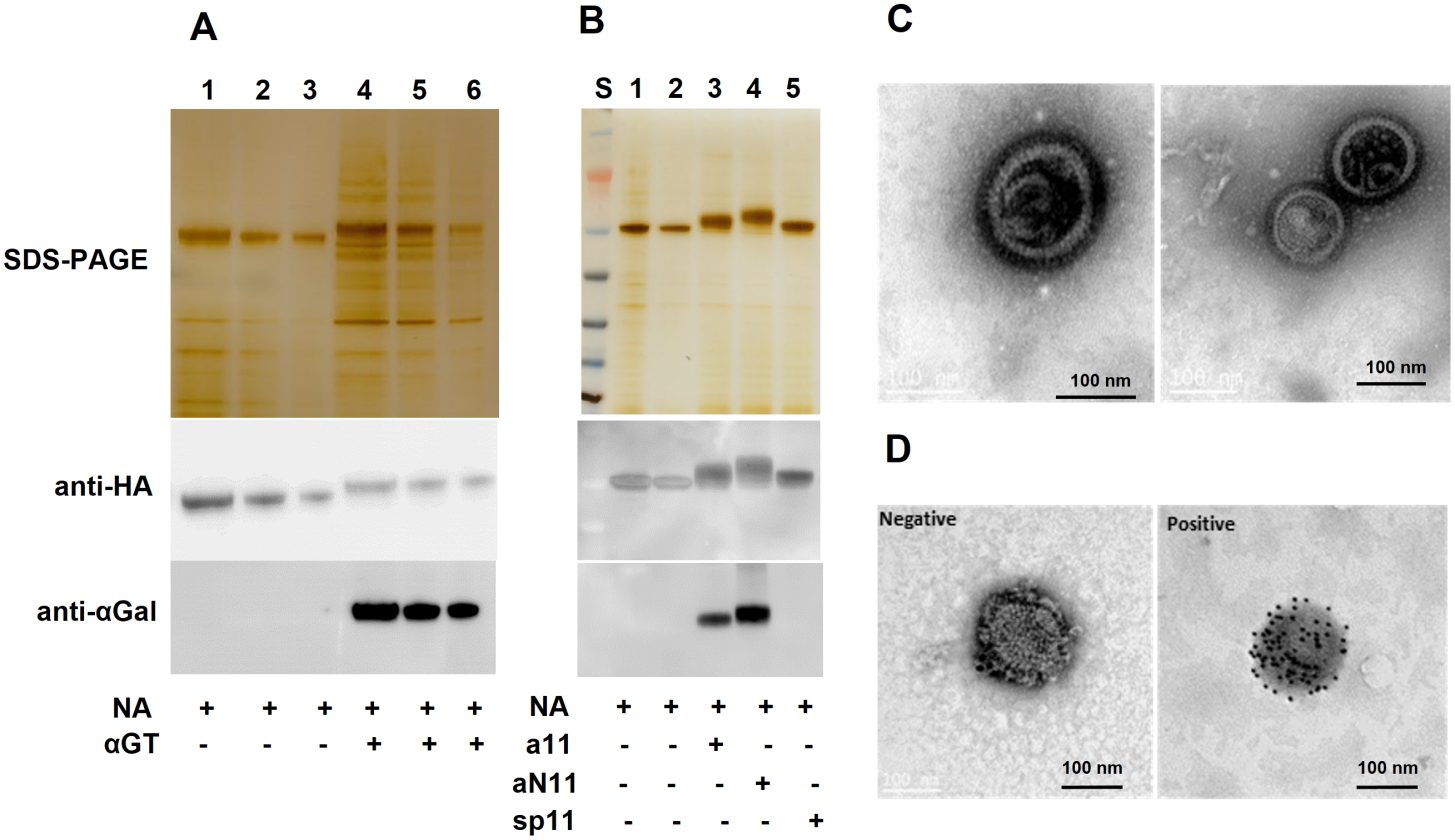
Biological and chemical modifications of H7N9 VLPs. (A) SDS-PAGE and western blot analysis of biologically modified H7N9 VLPs. Lane 1–3 represent 320 ng, 180 ng and 90 ng of αGal^(-)^ VLP based on HA content, respectively. Lane 4–6 are 130 ng, 90, and 40 ng of αGal^(+)^ VLP, respectively. (B) SDS-PAGE and western blot analysis of chemically modified H7N9 VLPs. S: protein standards; 1: αGal^(-)^ H7N9 VLP; 2: intermediate of αGal^(-)^ H7N9 VLP reacted with GO; 3: H7N9 conjugated with αGal linker a11; 4: H7N9 conjugated with αGal linker aN11; 5: H7N9 VLPs conjugated with control linker sp11. (C) Electron microscopy of H7N9 VLPs. (D) Electron microscopy of H7N9 VLPs stained with gold particles. Negative: VLPs were only stained with immunogold donkey anti-rabbit secondary antibody; Positive: VLPs were stained with anti-HA7 rabbit polyclonal antibody, followed by staining with immunogold donkey anti-rabbit secondary antibody. Sp11 is the aminooxy spacer control linker without αGal antigen; a11 is one type of αGal (Galα1,3-Galβ1,4-Glc) aminooxy linker; aN11 is the other type of αGal (Galα1,3-Galβ1,4-GlcNAc) aminooxy linker.

H7N9 VLPs were produced by co-transfecting codon optimized genes from the H7N9 virus (HA, NA and M1) using the previously published continuous harvest method [34, 35]. Biologically αGal-modified VLPs were generated by co-transfecting αGT gene with three influenza genes described above. The VLPs were purified by a series of ultra-centrifugation and centrifugation steps. Both anti-HA and anti-αGal western blot analyses verified that the dominant bands on the silver stained SDS-PAGE were HA proteins and αGT modified VLPs contained αGal epitopes (Fig 4A and 4B). Other genes, N9, M1 and αGT, which were not visualized on the SDS-PAGE, were also confirmed by corresponding western blot (data not shown). Electron microscopy (EM) experiments revealed the structural characteristics of H7N9 VLPs, which appeared as spherical enveloped particles with an average diameter of about 150 nm (Fig 4C). To specifically confirm the HA content in the particles, an H7-specific ImmunoGold staining experiment was performed. H7N9 VLPs were first incubated with anti-H7 primary antibody, followed by staining with a secondary antibody conjugated with gold particles (10 nm size). Electron microscopy was used to image the VLPs. The anti-HA (H7N9) antibody stained VLPs were identified with gold particles across the VLP surface (Fig 4D), while the control VLPs that were stained with the same secondary antibody only (no primary antibody) had no staining. These results indicated the successful production of H7N9 VLPs.

### Chemical modification of H5N1 and H7N9 vaccines

Recombinant protein and VLP vaccine candidates of H5N1 and H7N9 were chemically modified using our novel chemical modification (CarboLink) technology specifically targeting carbohydrates (Fig 2D). For each modification, a spacer that lacks the terminal carbohydrates was employed as a control linker (sp11). As shown in Figs 3B, 3C and 4B, vaccines modified by αGal aminooxy linkers (a11 or aN11) [32] were strongly stained with the anti-αGal antibody, but the samples linked with the control linker were completely negative by western blot. The size difference between vaccines before and after chemical linking was clearly visualized on SDS-PAGE, suggesting a significant number of αGal molecules were conjugated. All the modified vaccines retained hemagglutination activities (data not shown), indicating that our novel chemical modification method was compatible with the vaccines without disrupting their biological activities.

### Humoral immune response induced by αGal-modified H5N1 vaccines

**Fig 5:**
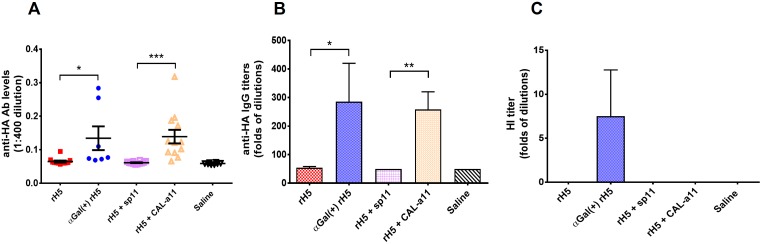
H5N1 HA specific antibody responses induced by αGal^(+)^ rH5 vaccines modified biologically and chemically. (A) anti-HA (H5N1) antibody responses from mice vaccinated with αGal-modified H5N1 HA or control vaccine at a dose 100 ng/injection. (B) anti-HA (H5N1) antibody end-point titers from mice vaccinated with αGal-modified H5N1 HA or control vaccine at 100 ng/dose. (C) HI antibody titer induced after H5N1 HA vaccination against H5N1 (A/duck/Vietnam/QT801/2011) HA. αGal(+) and CAL-a11 represent αGal biological modification and chemical modification, respectively. sp11 represents a negative control of chemical modification with the spacer only. Results are presented as mean with SEM. *p < 0.05, **p < 0.01, ***p < 0.001, ****p < 0.0001 for αGal positive versus αGal negative vaccines (by unpaired Student *t* test).

We initiated *in vivo* evaluation of H5N1 HA vaccine candidates without NA co-transfection in αGT knockout BALB/c mice. The mice were primed with an extract of pig kidney membranes (PKM) [36], incomplete Freund’s adjuvant (IFA) and a stimulatory CpG oligonucleotide before vaccination to generate elevated anti-αGal antibody levels. We found that this combination allows for effective class switching and induces multiple types of anti-αGal immunoglobulin, mimicking the strong pre-existing immunity of αGal in human (data not shown). Mice were then injected twice with the indicated vaccine 28 days apart. Blood was collected 14 days after the second vaccination from which serum was obtained for analysis. First, we examined the immune response against αGal-modified recombinant H5N1 HA (rH5) vaccines generated using both biological and chemical methods. The ELISA analysis using H5-HA as the coating antigen showed that biological and chemical αGal modifications were able to induce significantly higher anti-HA antibody titers compared to the un-modified version at 100 ng per dose (Fig 5A). Endpoint anti-HA antibody titer analysis confirmed the significantly enhanced immunogenicity of both αGal modification strategies (Fig 5B).

**Fig 6:**
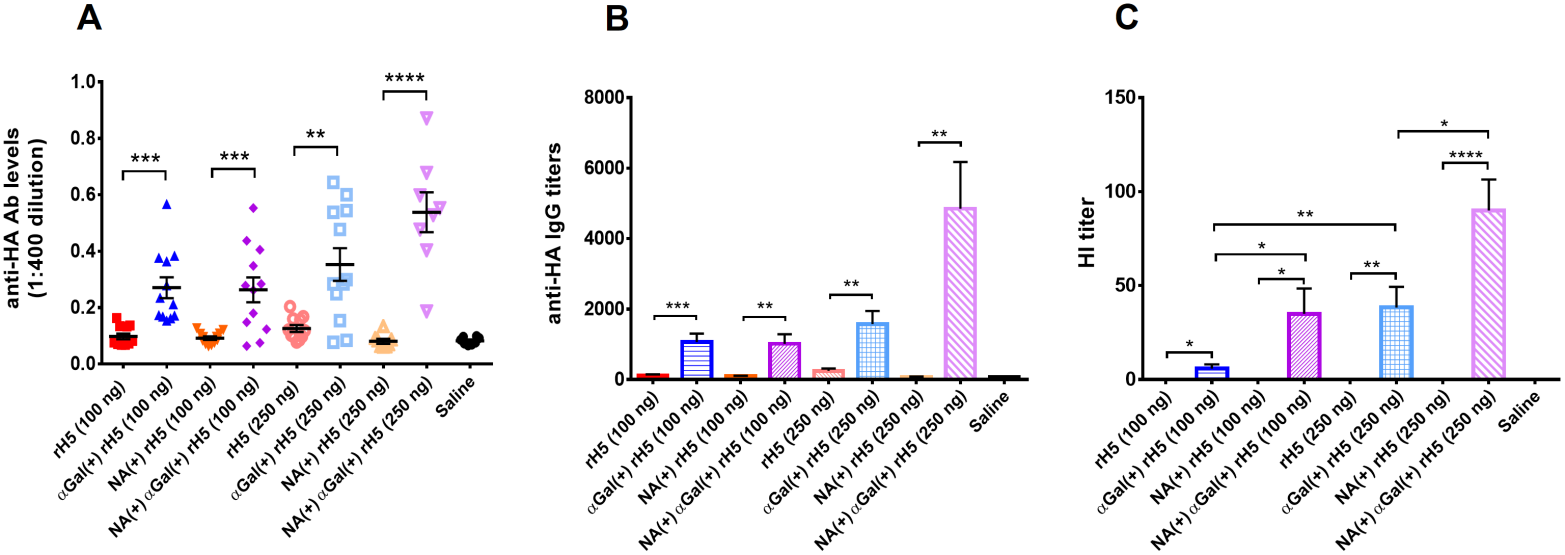
H5N1 HA specific antibody responses induced by αGal^(+)^ rH5 vaccines in the presence and absence of NA. (A) anti-HA (H5N1) antibody responses from mice vaccinated with αGal-modified H5N1 HA or control vaccine at 100 and 250 ng/dose. (B) End-point titer of anti-HA (H5N1) antibody from mice vaccinated with αGal-modified H5N1 HA or control vaccine at 100 and 250 ng/dose. (C) HI antibody titer induced after H5N1 HA vaccination against H5N1 HA (A/duck/Vietnam/QT801//2011). End-point OD value was defined as 3 times signal of background noise. Results are presented as mean with SEM. *p < 0.05, **p < 0.01, ***p < 0.001, ****p < 0.0001 for αGal positive versus αGal negative vaccines (by unpaired Student *t* test).

In order to confirm whether the induced anti-HA antibody level correlated with protection, hemagglutination-inhibition (HI) assay was performed, as HI antibody titer has been considered as the primary immune correlate of protection for influenza in many clinical studies[37–39]. In this study, we investigated the correlation between total anti-HA antibody from ELISA experiment and HI specific antibody from HI assay for our vaccination experiment. Surprisingly, low HI titers (HI < 20) were observed for biologically αGal-modified vaccine candidate only (Fig 5C). Despite of significantly higher antibody levels from both αGal-modified vaccines comparing to control group, neither of them were likely to elicit protection against potential H5N1 influenza infection at 100 ng/dose suggested based on their low HI titers. In the next experiment, we investigated dose response from slightly different rH5 vaccines at 100 ng/dose and 250 ng/dose (Fig 6). Interestingly, mice given 100 ng/dose NA^(+)^ αGal^(+)^ rH5 vaccine produced much higher HI titer than ones given the same dose of αGal^(+)^ rH5 vaccine (shown in Fig 6C), despite of similar anti-HA antibody levels (Fig 6A and 6B). When the vaccine dose was increased to 250 ng per vaccination, both αGal^(+)^ vaccines with and without NA co-transfection induced significantly increased HI titers (Fig 6C). Consistently, the rH5 vaccine produced in the presence of NA stimulated much stronger immunogenicity as assessed by hemagglutination activity (data not shown here) and hemagglutination inhibition assays. These results not only demonstrate that the αGal epitopes significantly enhanced the immunogenicity of H5N1 influenza vaccines, but also indicated that NA played an important role in eliciting protective antibodies and co-transfecting NA in the vaccine production will potentially lead to a more potent vaccine.

### Humoral immune responses to αGal-modified H7N9 vaccines

After successful demonstration of the enhanced humoral immunity generated by αGal-modification of H5N1 rHA vaccines, we further investigated αGal HyperAcute^™^ Technology on another highly pathogenic influenza strain H7N9, which caused 602 cases of human infection with 227 deaths reported from Mar 2013 to Feb 2015 [40–43].

**Fig 7:**
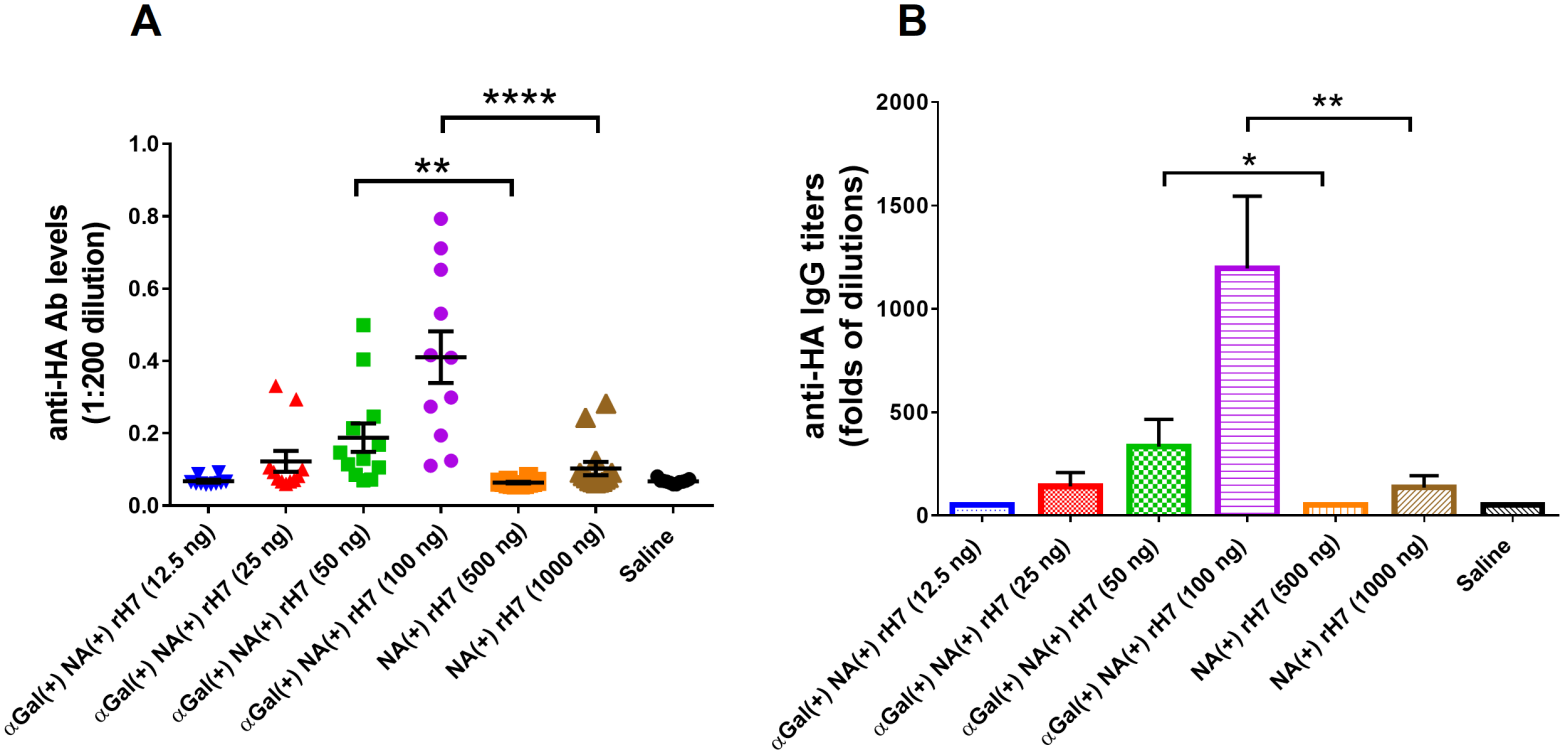
H7N9 HA specific antibody responses induced by αGal^(+)^ rH7 vaccines that were biologically modified. (A) anti-HA (H7N9) antibody responses from mice vaccinated with αGal-modified H7N9 HA or control vaccine at different doses. (B) End-point titer of anti-HA (H7N9) antibody from mice vaccinated with αGal-modified H7N9 HA or control vaccine at 100 and 250 ng/dose. End-point OD value was defined as 3 times signal of background noise. Results are presented as mean with SEM. *p < 0.05, **p < 0.01, ***p < 0.001, ****p < 0.0001 for αGal positive versus αGal negative vaccines (by unpaired Student *t* test).

A dose titration experiment of αGal^(+)^ and αGal^(-)^ H7N9 vaccines was performed in αGal knockout BALB/c mouse model. As the results show in Fig 7A and 7B, the αGal^(+)^ vaccines administered at 50 and 100 ng/dose elicited much higher anti-HA antibody levels than αGal^(-)^ versions even at 10-fold higher dose, 500 and 1000 ng/dose. These results clearly illustrate that αGal modification allows for a reduction of vaccine dose by at least 10-fold while still induces a strong immune response against H7N9 HA antigen.

**Fig 8:**
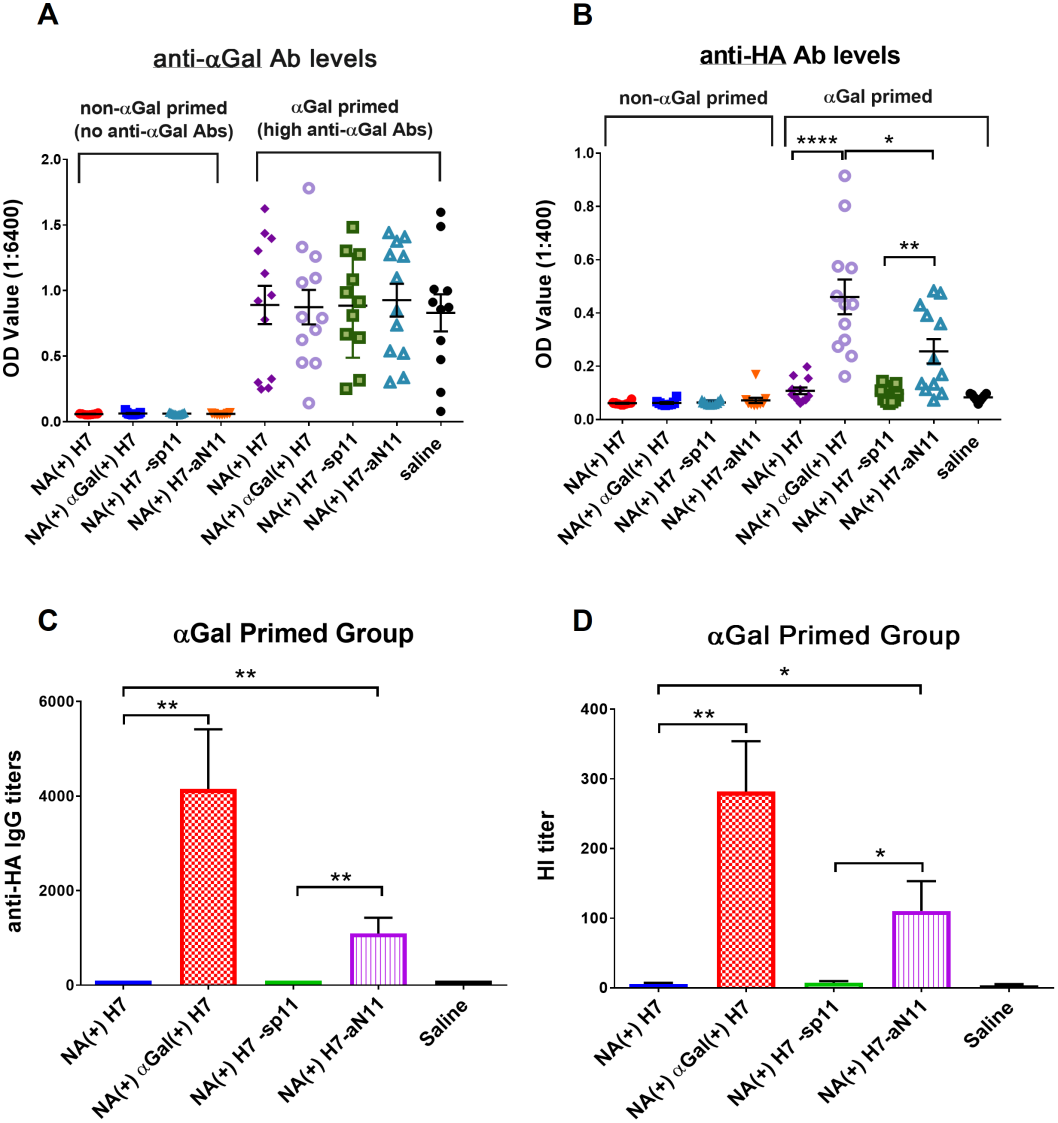
Comparison of H7N9 HA specific antibody responses induced by αGal^(+)^ rH7 vaccines in αGal primed with un-primed mice. (A) anti-αGal antibody responses from mice with or without αGal priming before H7N9 HA vaccination. (B) anti-HA (H7N9) antibody levels from mice vaccinated with αGal-modified H7N9 HA or control vaccine at 100 ng/dose. (C) End-point titer of anti-HA (H7N9) antibody from mice vaccinated with αGal-modified H7N9 HA or control vaccine at 100 ng/dose in the αGal primed group. (D) HI antibody titer induced after H7N9 HA vaccination against H7N9 HA in the αGal primed group. αGal(+) and aN11 refers to αGal biological modification and chemical modification, respectively. Results are presented as mean with SEM. *p < 0.05, **p < 0.01, ***p < 0.001, ****p < 0.0001 for αGal positive versus αGal negative vaccines (by unpaired Student *t* test).

In order to confirm that enhanced immunogenicity of vaccines was directly associated with anti-αGal Ab levels, αGal knockout mice which had not been primed, and thus should not have anti-αGal Abs were compared. As shown in Fig 8A, the group of αGT knockout mice without priming had no detectible anti-αGal Ab (non-αGal primed), while mice that were primed had high levels of anti-αGal Ab (αGal primed) even at the dilution of 1:6,400. The αGal-primed mice were grouped so that the mean anti-αGal antibody level was similar for each vaccine group. Mice were subcutaneously administered with two doses of 100 ng either biologically or chemically modified rHA vaccines, along with control vaccines. Both αGal-modified vaccines induced a strong humoral response against H7N9 HA compared with un-modified vaccines (Fig 8B) in mice with high anti-αGal Ab titers. It was noted that biologically modified rH7 vaccine elicited a stronger antibody response than chemically-modified vaccine in this experiment. As expected, there was no anti-HA Ab response from the group that was not primed, regardless of whether the vaccines were αGal positive or negative. In addition, hemagglutination inhibition titers further confirmed that pre-existing high anti-αGal antibody levels were absolutely required for αGal-modified vaccines to induce anti-HA antibodies (Fig 8C and 8D).

The striking difference in the antibody response between primed and un-primed mice confirmed that the immune-complex formed between the αGal epitope on the αGal-modified vaccine and anti-αGal Ab facilitated the presentation of the vaccine antigen by APC [19], which consequently enhanced the immune responses against the target antigen. This experiment demonstrated the potential of αGal HyperAcute^™^ technology in the vaccine development for human, who naturally have high pre-existing anti-αGal antibody level.

**Fig 9:**
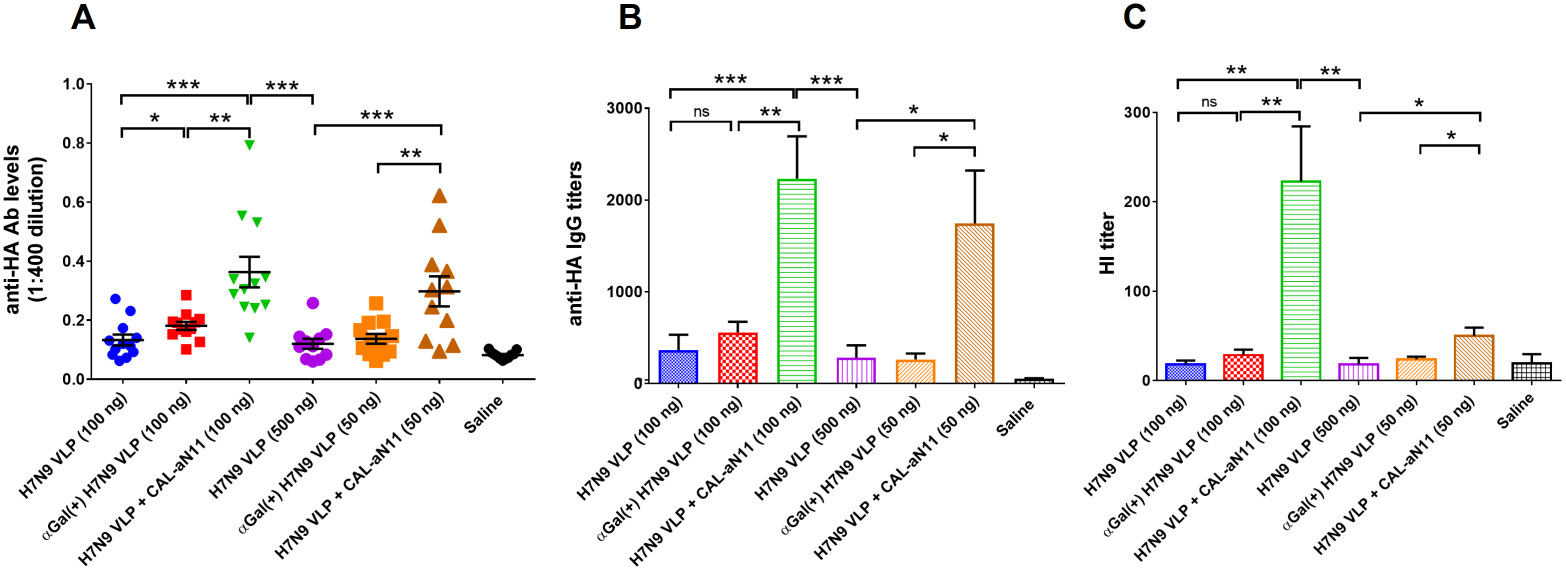
H7N9 HA specific antibody responses induced by αGal^(+)^ VLP vaccines. (A) anti-HA (H7N9) antibody responses from mice vaccinated with αGal-modified H7N9 VLP at different doses. (B) End-point titer of anti-HA (H7N9) antibody from mice vaccinated with αGal-modified H7N9 VLP or control vaccine at different doses. (C) HI antibody titer induced after VLP vaccination against H7N9 (A/Hangzhou/1/2013) HA. αGal(+) and CAL-aN11 refer to αGal biological modification and chemical modification, respectively. Results are presented as mean with SEM. ns: not significant, *p < 0.05, **p < 0.01, ***p < 0.001, ****p < 0.0001 for αGal positive versus αGal negative vaccines (by unpaired Student *t* test).

In addition to H7N9 recombinant HA vaccine candidates, we have also investigated VLP platform. As shown in Fig 9, both biologically and chemically modified αGal^(+)^ VLP vaccines elicited significant anti-HA antibody response when compared with un-modified control groups (Fig 9A). Interestingly, VLP vaccines that were chemically modified (only data of CAL-aN11 linker was shown) induced much higher anti-HA antibody levels and HI titers than biologically modified VLPs (Fig 9B and 9C). One possible reason is that our chemical modification technology modified all antigens on the VLP surface, including not only HA, but also NA, and any other existing surface antigens, such as glycolipids. The overall higher level of αGal epitopes on chemically modified VLP surface may have led to a higher anti-HA response. Again, the significant difference between 50 ng/dose of αGal-modified VLP (H7 VLP + CAL-aN11) and the 500 ng/dose of un-modified version (H7 VLP) indicated that the αGal technology improved the potency of H7N9 VLP vaccine candidates at least 10-fold in mice.

### Correlation between the level of αGal epitopes and vaccine immunogenicity

**Fig 10:**
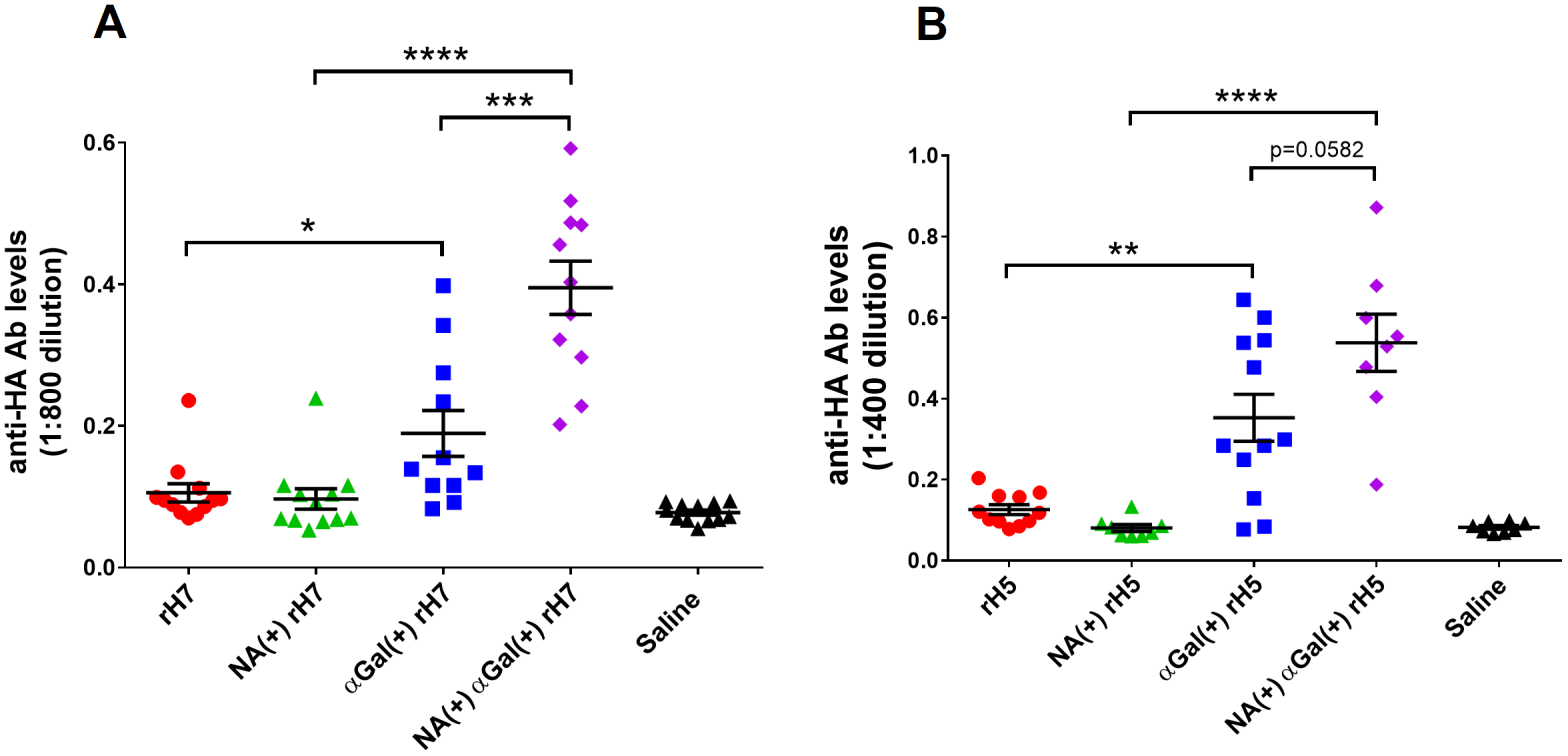
H5N1 and H7N9 HA specific antibody responses induced by αGal^(+)^ rHA vaccines produced in the presence or absence of NA. (A) anti-HA (H7N9) antibody responses from mice vaccinated with biologically αGal-modified H7N9 HA or control vaccine at 100 ng per dose. (B) anti-HA (H5N1) antibody titers from mice vaccinated with biologically αGal-modified H5N1 HA vaccine or control vaccine at 250 ng per dose. NA(+) represents the NA co-transfected in the vaccine production process. Results are presented as mean with SEM. ns: not significant, *p < 0.05, **p < 0.01, ***p < 0.001, ****p < 0.0001 for αGal positive versus αGal negative vaccines (by unpaired Student *t* test).

**Fig 11:**
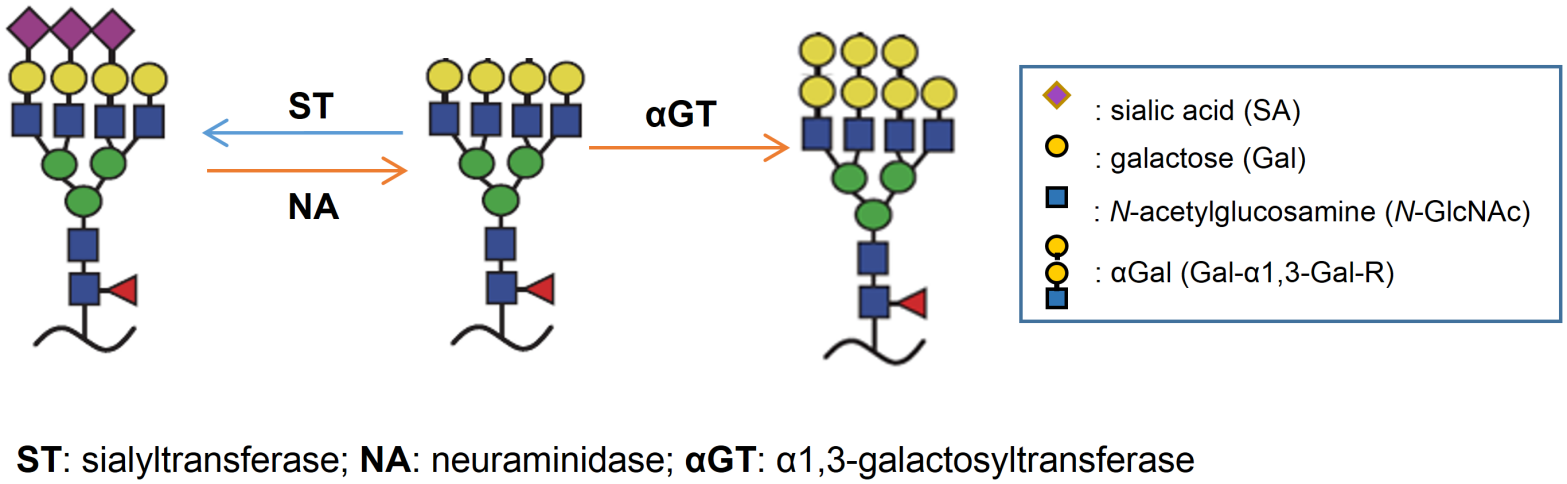
Illustration of last step of *N*-glycosylation biosynthetic pathway in mammalian cell expression system. Sialyltransferase (ST) transfers sialic acid to terminal galactose on oligosaccharide. There is a competition between ST and αGT in the absence of NA, since αGT utilizing the same intermediate as for ST. This competition can be biased in the presence of NA, which reverses the reaction catalyzed by ST, and the formation of αGal is irreversible.

To understand the role of NA in stimulating humoral immune response, we conducted an experiment to compare the anti-HA antibody level induced by αGal^(+)^ vaccines that were produced in the presence or absence of NA. As shown in Fig 10A, The difference between anti-H7 response stimulated by αGal^(+)^ rH7 vaccines that were produced in the presence of NA (αGal^(+)^NA^(+)^ rH7) and absence of NA (αGal^(+)^ rH7) was quite striking with a p-value of 0.005. We also examined H5N1 rHA vaccines that were similarly prepared (Fig 10B). Consistently, NA co-expressed αGal^(+)^ rH5 vaccine induced better humoral immune response against H5N1 antigen at 250 ng/dose with a p-value of 0.0582. NA is an enzyme that cleaves terminal sialic acid molecules, in turn generating substrates that are available for αGal modification by αGT. It clearly plays a crucial role in stimulating higher humoral response. One hypothesis is that NA the different potency of αGal-modified vaccines in the presence or absence of NA is likely related to the degree of αGal modification (Fig 11).

In order to verify that the presence of NA makes a difference in numbers of sialic acid molecules, we performed a sialic acid quantification assay on purified rHA products with or without NA co-transfection. This assay served as an indirect method to analyze the amount of αGal modification. As illustrated in Fig 11, the more sialic acid removed by NA, the more αGal that can be generated by αGT. As shown in Table 1, rH5 alone had 28 sialic acid molecules on average on each HA molecule, but only 11 in the presence of αGT. Presumably, there were 17 αGal generated by αGT, while there were still 11 sialic acid molecules remaining as a result of competition between αGT and sialyltransferase. In the presence of NA, the number of sialic acid molecule was further reduced to 2, which suggested that 26 out of original 28 sialic acids were removed by NA, and consequently resulted in 26 sites available for αGal modification. A similar result was observed in rH7 vaccines, suggested by the increased number of exposed sites for αGal modification (40 in the presence of NA and 28 in the absence of NA). It was observed that αGal^(+)^N9^(+)^ rH7 contained slightly more sialic acid molecules than αGal^(+)^N1^(+)^ rH5, presumably either due to the yields from both NA and αGT, or different enzymatic activity between N1 and N9. By subtraction, we can approximately derive the number of αGal modification per HA molecule. As shown in the last column, significantly more αGal epitopes were added per HA when the NA was co-expressed. Interestingly, there were more αGal epitopes formed on rH7 than on rH5. This ratio correlated to about 2-fold difference in the minimum dose which was required to elicit strong immune response. As low as only 100 ng per dose of αGal^(+)^ rH7 induced strong antibody titer (Fig 10A), while 250 ng per dose was required for αGal^(+)^ rH5 to see a similar response (Figs 6 and 10B). This approximate 2-fold difference in the minimal dose was well correlated to the 2-fold difference in the amount of potential αGal modification between rH5 and rH7 vaccines, in the absence or presence of NA co-transfection.

**Table 1:** Sialic acid quantification assay.

Vaccines	Sialic acid/HA	Potential total # of αGal modification sites	Derived αGal/HA	Additional αGal/HA (Theoretical) due to NA co-expression
rH5	28	28		**9**
αGal(+) rH5	11	28	28–11 = **17**	
NA(+) rH5	1	28		
αGal(+) NA(+) rH5	2	28	28–2 = **26**	
rH7	48	48		**12**
αGal(+) rH7	20	48	48–20 = **28**	
NA(+) rH7	7	48		
αGal(+) NA(+) rH7	8	48	48–8 = **40**	

The numbers of sialic acid on different HA represent the numbers of sialic acid remaining on each HA molecule. The derived αGal/HA based on the assumption that all the removed sialic acid sites can be modified by αGT. Practically, the results represent the relative αGal modification ratio under different conditions.

### Comparison of αGal with traditional adjuvants on vaccine immunogenicity

**Fig 12:**
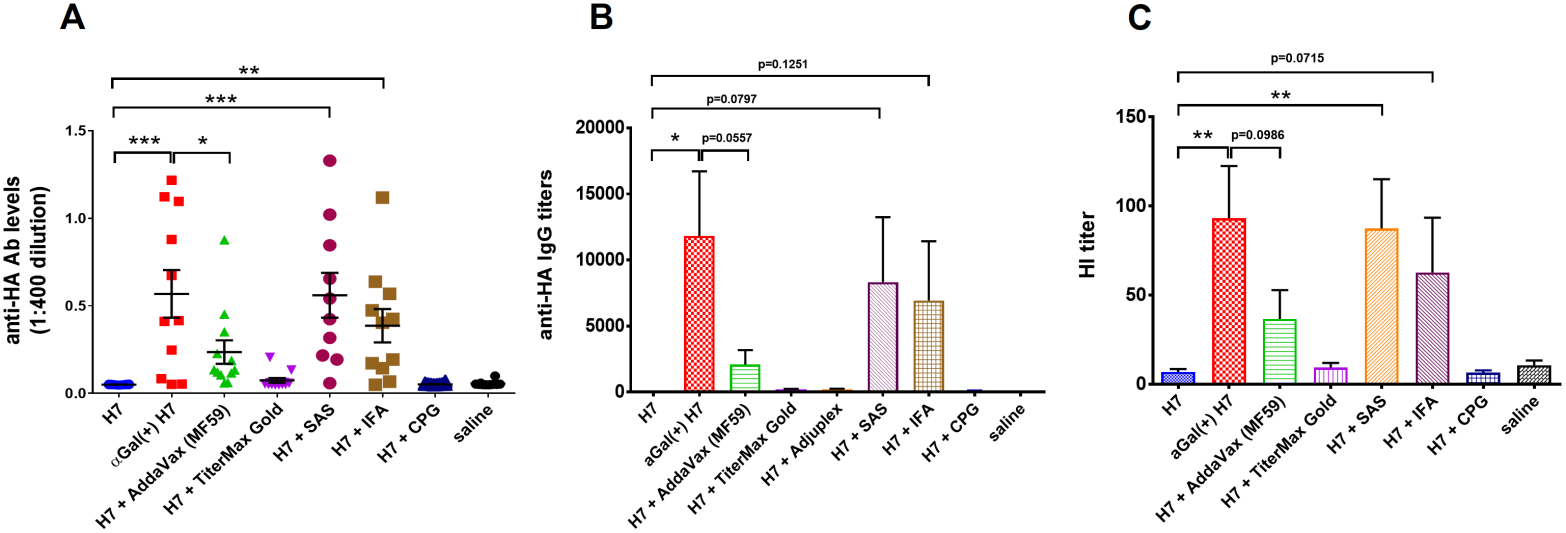
H7N9 HA specific antibody responses induced by rH7 vaccines with αGal or traditional adjuvants. (A) anti-HA (H7N9) antibody responses from mice vaccinated with H7N9 HA at 100 ng per dose with αGal or traditional adjuvants. (B) End-point titer of anti-HA (H7N9) antibody from mice vaccinated with differently formulated vaccines. (C) HI antibody titer induced after H7N9 HA vaccination against H7N9 (A/Hangzhou/1/2013) HA. Results are presented as mean with SEM. *p < 0.05, **p < 0.01, ***p < 0.001, ****p < 0.0001 for αGal positive versus αGal negative vaccines (by unpaired Student *t* test).

Although the demand for commercial vaccines against pandemic avian influenza has become more urgent since the emergence of H7N9 in 2013, the development of effective avian influenza vaccines has been hampered by the poor immunogenicity of vaccine candidates [44–46]. Currently, the only approved avian influenza vaccine in US is AS03-adjuvanted H5N1 influenza vaccine, which is stockpiled in case of pandemic outbreak. In addition, several water-in-oil adjuvanted H7N9 vaccine candidates are currently under clinical evaluations. In order to further understand the potential of our αGal HyperAcute^™^ technology, we conducted a mouse experiment to compare αGal induced immunity with several traditional adjuvants, including MF59, IFA and CpG. A group of αGT Knockout mice were subcutaneously vaccinated with H7N9 HA vaccines without αGal, with αGal, or with different adjuvants (using the recommended dose by the manufacturers), following the same protocol established for our previous immunization experiments. Consistently, αGal positive vaccine elicited strong humoral immune response against H7N9 HA (Fig 12A and 12B). Among the adjuvanted vaccine groups, the most promising adjuvant MF59 that is in clinical trials, only showed moderate immune enhancement with low levels of antibody observed. Both Sigma adjuvant system (SAS) and incomplete Fruend’s adjuvant (IFA) also induced strong humoral immunity against H7N9 viral glycoprotein, with a level very close to un-formulated αGal^(+)^H7. These adjuvants resulted in adverse skin reactions in approximately 10% of mice injected with the vaccine, but in no cases have we observed an adverse reaction with αGal-modified flu vaccine. Nevertheless, these two adjuvants are unlikely to be introduced in human influenza vaccines. Furthermore, HI titers confirmed that high anti H7N9 HA antibody levels well correlated with hemagglutination inhibition (Fig 12C), which suggests the protective humoral immunity induced by the vaccine candidates. The results strongly suggest the potential of αGal technology in developing potent avian influenza vaccines.

## Discussion

In 2013, an outbreak of avian influenza A (H7N9) virus posed a pandemic threat for human health. We initiated the development of potent vaccines against two highly pathogenic avian influenza strains, H5N1 and H7N9, using αGal HyperAcute^™^ technology. We established two αGal modification strategies and successfully produced recombinant HA and VLP vaccine candidates that were modified with αGal epitopes using either biological or CarboLink modification techniques. For biological modification, the recombinant HA protein or VLP over-expressed in HEK293F cells were post-translationally modified with αGal by co-transfecting the αGT gene along with genes for the vaccine. This type of modification generates αGal at the last step of N-glycan biosynthesis pathway in Golgi [47], where natural N-glycosylation takes place. Therefore, it poses minimal effect on the native structure and function of the proteins. However, its application is limited to glycoprotein targets that are expressed in mammalian cells and contain N-linked glycosylation sites. An alternative way to obtain this result is to biochemically introduce αGal using αGT enzyme and UDP-Gal[13, 48]. However, the high cost and laborious process for manufacture of both αGT enzyme and UDP-Gal restrict the potential of this strategy in vaccine production.

To broaden the application of αGal HyerAcute^™^ technology, we developed two chemical modification methods to conjugate αGal molecules on αGal^(-)^ vaccine products using αGal chemical linker molecules with key functional groups. The first method uses an αGal NHS linker (Fig 2C), which adds αGal epitopes to lysine residues on the protein. Although this lysine-specific modification strategy has high potential to modify any target proteins and is able to conjugate relatively large amount of αGal epitopes on the target, there are concerns on changing the physical and biological activities of the antigens. In addition, receptor-binding domains (RBD) on lysine-rich proteins might be blocked due to undesired modification on lysine residues in this region. The second method, CarboLink, specifically installs αGal molecules onto existing glycosylation sites without interfering with amino acid residues. This carbohydrate-specific modification strategy allows αGal-modified products to closely mimic biologically modified molecules. Another advantage is the broader application to any type of glycoprotein. The hemagglutination assay results indicated that the CarboLink strategy was able to generate influenza vaccines with intact biological activity, while the lysine-specific modification resulted in the loss of the hemagglutination activity, presumably by blocking the RBD.

Anti-HA antibody titers and HI titers of the mice immunized with αGal^(+)^ vaccines demonstrated that both biologically and chemically αGal-modified vaccines stimulated strong immune response in αGal knock-out mice with two vaccinations at a dose of as low as 100 ng for H7N9 vaccine and 250 ng for H5N1 vaccine in either recombinant HA or VLP platforms. Our results indicate that αGal modifications significantly enhanced the immunogenicity of the influenza vaccines in terms of humoral responses and αGal-modified products were at least 10-fold more potent when compared with regular vaccines without αGal modification. In other words, αGal technology significantly reduced the required vaccination dose at least 10 fold while retaining desired immunogenicity. It should also be noted that given the highly efficacious nature of the modification, smaller mouse groups can be used in this model system. This advance has the potential to make vaccine production more cost-effective due to dose-sparing, leading to a more affordable product for the end user worldwide. It is also beneficial to meet the demand in the case of emerging infectious disease outbreak in human vaccine. Furthermore, our low effective dose of 100 ng is remarkable as compared with the doses used in most of the current pre-clinical influenza vaccine studies, which normally ranged from 1 μg to 50 μg per injection per mouse injection [19, 49, 50], in split-virion, VLP or inactivated virus vaccines used in other studies. Additionally, there was no traditional adjuvant needed for our novel αGal-modified recombinant influenza vaccines, while adjuvants were usually necessary in most of studies in order to achieve statistical significance, particularly in the cases of H5N1 and H7N9 influenza vaccine development [41–43, 51]. We note that this system could also be used for avian vaccines as well.

During the correlation study between αGal epitopes and vaccine immunogenicity, we discovered that NA plays a critical role in the HyperAcute-based vaccine production process. Our investigation leads to a conclusion that the NA helps to increase the degree of αGal modification. It is well known that mammalian N-glycosylation biosynthetic pathway furnishes the glycosylation site with terminal sialic acids on a galactose molecule by sialyltransferase *via* an α2,3- or α2,6- O-glycosidic linkage [52]. In the absence of NA, co-expressed αGT introduces competition between sialyltransferase and αGT in the last step of glycosylation pathway (Fig 11), since both of these enzymes target the same terminal galactose substrates. As a result, there is equilibrium between generating glycoproteins bearing terminal sialic acid and αGal. Therefore, both sialic acid positive and αGal positive products co-exist under this condition. However, in the presence of NA, the glycosylation reaction favors αGT pathway, as the terminal sialic acids introduced by sialyltransferase are hydrolyzed by NA to push the reaction towards terminal αGal. In the other words, this condition reverses the reaction of sialyltransferase, and thus renders more terminal galactose substrates for αGT modification. Consequently, more αGal epitopes are ultimately generated on the glycosylation sites with NA co-expression than without NA. Our study revealed that more αGal epitopes per vaccine molecule lead to stronger immunogenicity.

In the comparison study between αGal and traditional adjuvants, αGal technology demonstrates effective enhancement of vaccines efficacy. Remarkably, the αGal modifications on H7N9 vaccine elicited significantly higher antibody response than MF59 adjuvant, which has been approved to be used as an adjuvant in seasonal influenza vaccine outside the US. In our study, αGal was shown to be as effective as two other adjuvants, SAS and IFA, both of which were considered as powerful immunostimulants that have been exclusively optimized for use in animal models. Since our αGal technology introduces αGal epitope on vaccine candidates *via* covalent bonds, the manufacturing process will be simpler and much more efficient in that it obviates formulation and QA/QC issues encountered with most of the traditional adjuvants. In addition, a stability study has demonstrated that both biologically and CarboLink modified vaccine candidates can be stored at 4°C for at least 1 year in the form of lyophilized powder (data not shown).

Our success in developing highly potent H5N1 and H7N9 vaccine candidates demonstrated the great potential of αGal technology in the development of vaccines against other infectious disease agents. Although it would be more informative if viral neutralization and mouse challenge data were available, we were not able to pursue these studies as handling H5N1 and H7N9 virus requires biological safety level 3 (BSL-3) containment that were not available for our studies. However, we were able to demonstrate thatαGal-modified H1N1 vaccine is significantly more efficacious against H1N1 PR8 virus challenge after the prime-boost vaccinations relative to unmodified vaccine (manuscript in preparation).

## Supporting information

S1 File Methods for protein and virus-like particle (VLP). This file contains the details of the techniques used to express and purify the recombinant proteins and VLPs used for testing the efficacy of αGal modification.
(DOCX)

S2 File Chemical modification methods. The file contains the details of the techniques used to chemically modify the proteins and VLPs with αGal.
(DOCX)

S3 File Raw data for figures. This spreadsheet contains the raw data for Figs 5, 6, 7, 8, 9, 10 and 12 as a separate tab in the file.
(XLSX)
