# Supplementary material for: Addition of αGal HyperAcute™ technology to recombinant avian influenza vaccines induces strong low-dose antibody responses
Source: PLoS One. 2017 Aug 7;12(8):e0182683. doi: 10.1371/journal.pone.0182683 (PMC5546595; doi:10.1371/journal.pone.0182683)
Supplement: S1 File — This file contains the details of the techniques used to express and purify the recombinant proteins and VLPs used for testing the efficacy of αGal modification. (DOCX) [file pone.0182683.s001.docx]

## **S1 File. Recombinant protein and virus-like particle (VLP) expression and purification.** This file details the methods used to express and purify recombinant protein and VLPs utilized to measure the immune response to αGal modified vaccines.

**Recombinant protein expression and purification**

For both H5N1 and H7N9 HAs, four versions of the protein were produced: HA, NA^(+)^HA, αGal^(+)^HA, and NA^(+)^αGal^(+)^HA. Genes of these proteins were transiently co-transfected into HEK293F cells (Invitrogen, R790-07) maintained in FreeStyle 293 Expression Medium (Life Technologies, 12338-018). For each liter of cell culture, 1 mg of total DNA (HA only, HA to NA [3:1], HA to αGT [3:1], or HA to NA to αGT [3:1:1]) was mixed with 3 mg PEI-MAX (Polysciences, 24765-2). The mixture was incubated at room temperature for 40 minutes before adding to 10^9^ cells. Transfected cells were incubated on an orbital shaker platform rotating at 125 rpm in a 37 °C incubator with 80% humidity and 5% CO_2_. Each liter of culture was supplemented with sodium butyrate (1 mg) 24 hour post-transfection. The secreted protein was expressed for three days before harvest.

The supernatant of cell culture was harvested, filtered, and then concentrated using tangential flow filtration (TFF) through a Pellicon 2 Mini Cassette (Millipore, P2B100C01). The protein was purified using immobilized metal affinity chromatography (GE Healthcare, 17-3712-05). The purified protein was quantified using a biscinchinonic acid (BCA) protein assay kit (Thermo Scientific, 23225). The plate was read using EPOCH plate reader (BioTek) with Gen5 software.

## **Recombinant Virus-Like Particle (VLP) Expression and Purification**

HEK293F suspension cells were maintained in EX-CELL 293 media (Sigma-Aldrich, 14571C) with 4 mM L-glutamine and 50 Unit/mL Penicillin-Streptomycin (Life Technologies, 15140-122). Before transfection, HEK-293F cells were centrifuged at 300 *x g* and re-suspended at 2 x 10^6^ cells/mL in fresh transfection medium, RPMI 1640 medium (Life Technologies, 21870-076) supplemented with 4 mM L-glutamine (Life Technologies, 25030-081), 5% FBS (Life Technologies, 16000-044), 25 mM HEPES (Life Technologies, 15630-080) and 1% Pluronic F68 (Life Technologies, 24040-032). For every 2 x 10^6^ cells, 2.5 μg influenza VLP plasmids HA:NA:M1:αGT or HA:NA:M1:pcDNA3 (3:1:3:1) were diluted in 50 μL of 150 mM NaCl and mixed with 7.5 μL of 1 mg/mL PEI diluted in 42.5 μL of 150 mM NaCl . The DNA-PEI mixture was incubated at room temperature for 8 minutes and then added to the cells. After 6 hours, a volume of EX-CELL 293 medium (supplemented with 4 mM L-glutamine) equivalent to the total volume of transfected culture was added to the transfected cells. Transfected cells were incubated on an orbital shaker platform rotating at 125 rpm in a 37 °C incubator with 80% humidity and 5% CO_2_.

Supernatant from the transfected cells was harvested every 12 hours for 96 hours, post-transfection, by centrifuging the cells at 300 *x g* for 3 minutes at 4 °C and collecting the supernatant. Cells were re-suspended in fresh EX-CELL 293 medium supplemented with 4 mM L-Glutamine. The harvested supernatant from each time point was pooled and clarified at 2000 *x g* for 15 minutes at 4 °C. NaCl was added to the clarified supernatant to a final concentration of 376 mM, and then the supernatant was 0.2 μm filtered. Samples were concentrated using TFF through a Pellicon 2 Mini Cassette. Additional purification and concentration of influenza VLPs was performed by 2,000 *x g* centrifugation for H7N9 VLPs. The VLP pellets were re-suspended in 4% sucrose (Fisher, S5-3) prepared in 1X DPBS (Lonza, 17-512F) and stored at -80 °C.
