# Supplementary material for: Addition of αGal HyperAcute™ technology to recombinant avian influenza vaccines induces strong low-dose antibody responses
Source: PLoS One. 2017 Aug 7;12(8):e0182683. doi: 10.1371/journal.pone.0182683 (PMC5546595; doi:10.1371/journal.pone.0182683)
Supplement: S2 File — The file contains the details of the techniques used to chemically modify the proteins and VLPs with αGal. (DOCX) [file pone.0182683.s002.docx]

**S2 File. Chemical modification methods.** Details of the procedures used to chemically modify influenza vaccine candidates with the αGal carbohydrate.

### **General procedure for preparation of immobilized galactose oxidase (iGO).**

One (1) mL of galactose oxidase (Worthington Biochemical Corp.) solution at 7.6 mg/mL was added to 1 g of dry NHS-activated agarose resin (ThermoFisher Sci., 26196) in a 15 mL spin column. After the mixture was mixed end-over-end for one hour at room temperature, the column was centrifuged at 1,000 *x* g for 2 minutes, and the flow-through was discarded. The resin was washed twice using 1X DPBS by centrifugation at 1,000 *x* g for 2 minutes each time, and the flow-through was discarded. Then 6 mL of 1 M Tris buffer pH 7.5 (Invitrogen 15567-027) was added to the resin column, and mixed end-over-end for 20 minutes at room temperature. The column was centrifuged at 1,000 *x* g for 2 minutes, and the flow-through was discarded. The resin was washed twice using 1X DPBS by centrifugation at 1,000 *x* g for 2 minutes each time, and the flow-through was discarded. 1X DPBS was added to the immobilized galactose oxidase product (iGO) to a final volume of 10 mL. The product was stored at 4 °C for up to 6 months. The enzyme activity of the iGO was determined to be approximately 30 U/mL by Amplex® Red Galactose/Galactose Oxidase Kit (Molecular Probes, A22179).

### **General procedure for αGal linker modification of recombinant glycoprotein**

1. Oxidation of glycoprotein by immobilized galactose oxidase (iGO).

One hundred (100) μL of iGO (30 U/mL) were added to 100 μL of glycoprotein (1 mg/mL) in 1X DPBS in a spin column. After incubation at 37 °C for 4 hours, the column was centrifuged at 1,000 *x* g for 2 minutes, and the flow-through was collected. The resin was washed twice using 1X DPBS by centrifugation at 1,000 *x* g for 2 minutes, and the flow-through was collected. The two flow-throughs were combined and concentrated by centrifuging at 14,000 *x* g for 10 minutes using a 10 kDa cut-off filter device (Millipore, UFC501096); the flow-through was discarded. The product was washed once with 0.4 mL of 0.1 M NaOAc buffer (pH 5.5) using the same filter device at 14,000 *x* g for 10 minutes. The final product was obtained as a 100 μL solution by adjusting the volume with 0.1 M NaOAc buffer (pH 5.5).

1. Conjugation with αGal aminooxy linker.

To 100 μL of oxidized glycoprotein solution from the oxidation of HA by iGO, 5 μL of αGal aminooxy linker (20 mg/mL) and 0.5 μL of aniline (Sigma, 242284) were added. The reaction mixture was shaken overnight at 4 °C and concentrated by centrifuging at 14,000 *x* g for 10 minutes using a 10 kDa cut-off filter device; the flow-through was discarded. The concentrate was washed twice using 1X DPBS. The final product was obtained as a 100 μL solution by adjusting the volume with 1X DPBS and stored at -20°C.

**General procedure for αGal linker modification of VLPs**

1. Oxidation of VLP by galactose oxidase (GO).

Ten (10) μL of catalase (10 U/μL, Sigma, C9322) and 5 μL of GO (500 U/ml; Sigma, G7907) were added to 170 μL of VLPs (300 ng/μL) in 1X DPBS. After incubation at 37 °C for 2 hours, the mixture was centrifuged at 21,000 *x* g for 30 minutes to pellet the VLPs and the supernatant was discarded. The pellet was re-suspended in 200 μL 1X DPBS and centrifuged again. The supernatant was discarded and the pellet was re-suspended with 150 µL of 0.1 M NaOAc buffer (pH 5.5).

1. Conjugation with αGal aminooxy linker.

To the 150 μL of oxidized VLP suspension from the oxidation of VLP by GO, 5 μL of αGal aminooxy linker (20 mg/mL) and 0.75 μL of aniline were added. The reaction mixture was shaken overnight at 4 °C, and then centrifuged at 21,000 *x* g for 30 minutes to pellet the VLPs. The supernatant was discarded and the pellet was re-suspended in 200 μL 1X DPBS. The wash with 1X DPBS was repeated twice. The final pellet was re-suspended in 80 μL of 4% sucrose prepared in 1X DPBS and stored at -20 °C.
